# Retraction Note: Effect of sagittal femoral component alignment on biomechanics after mobile-bearing total knee arthroplasty

**DOI:** 10.1186/s13018-021-02314-8

**Published:** 2021-03-10

**Authors:** Yong-Gon Koh, Jin-Ah Lee, Hwa-Yong Lee, Dong-Suk Suh, Hyo-Jeong Kim, Kyoung-Tak Kang

**Affiliations:** 1 Joint Reconstruction Center, Department of Orthopaedic Surgery, Yonsei Sarang Hospital, 10 Hyoryeong-ro, Seocho-gu, Seoul, 06698 Republic of Korea; 2 Department of Mechanical Engineering, Yonsei University, 50 Yonsei-ro, Seodaemun-gu, Seoul, 03722 Republic of Korea; 3 Department of Sport and Healthy Aging, Korea National Sport University, 1239 Yangjae-dearo, Songpa-gu, Seoul, 05541 Republic of Korea

**Retraction Note: J Orthop Surg Res 14, 400 (2019)**

**https://doi.org/10.1186/s13018-019-1458-5**

The Editor-in-Chief has retracted this article because it contains material that substantially overlaps with the following works (among others) [1–4].

Jin-Ah Lee does not agree to this retraction. Yong-Gon Koh, Hwa-Yong Lee, Dong-Suk Suh, Hyo-Jeong Kim & Kyoung-Tak Kang have not responded to any correspondence from the publisher about this retraction.
